# Evaluation of *Octopus maya* enzyme activity of the digestive gland and gastric juice

**DOI:** 10.1242/bio.060429

**Published:** 2024-09-12

**Authors:** Daisy Pineda-Suazo, Wendy Escobedo-Hinojosa, Lenin E. Fabian-Canseco, Pedro Gallardo, Cintia Moguel-Ojeda, Claudia Caamal-Monsreal, Ariadna Sánchez-Arteaga, Carlos Rosas

**Affiliations:** ^1^ Unidad Multidisciplinaria de Docencia e Investigación, Facultad de Ciencias UNAM, Puerto de abrigo s/n Sisal, Mpio, Hunucma, Yucatán, C. P. 97356, México; ^2^ Unidad de Química en Sisal, Facultad de Química, Universidad Nacional Autónoma de México, Puerto de abrigo s/n, 97356 Sisal, Yucatán, México; ^3^ Instituto Tecnológico Superior de Tlatlauquitepec, Carretera Federal Amozoc-Nautla Km. 122+600 Almoloni Tlatlauquitepec, Puebla, C. P. 73907, México; ^4^ Posgrado en Ciencias del Mar y Limnología, Facultad de Ciencias, Universidad Nacional Autónoma de México, Puerto de abrigo s/n, Sisal, Yucatán, C. P. 97356, México

**Keywords:** *Octopus maya*, Digestive gland, Enzyme activity, Gastric juice, Cephalopod digestion

## Abstract

As the demand for *Octopus maya* grows, sustainable farming practices become essential to prevent overexploitation, so that farming can be developed as a sustainable alternative to traditional fishing. Understanding the digestive dynamics of the octopus is essential for devising optimal dietary formulations in aquaculture. Despite the progress in understanding cephalopod digestion, little is known about the specific functioning of the digestive enzymes responsible for breaking down protein substrates. This knowledge gap underscores the need for further research to support sustainable *O. maya* population management. In this paper, dietary formulations are identified for cephalopods by characterizing *O. maya* digestive enzymes present in the digestive gland and gastric juice. The investigation revealed that acidic proteases showed a peak activity at higher temperatures than alkaline proteases. Inhibitors confirmed the presence of H, L, and D cathepsins. The lower activation energy of alkaline enzymes compared to acidic ones observed highlights an intriguing aspect of *O. maya's* digestive physiology. This research provides valuable insights into *O. maya* digestive enzyme functions, representing a significant advancement in formulating diets crucial for successful octopus farming that may help to fully understand its physiology.

## Teaser

**Summary:** This research provides valuable insights into *Octopus maya* digestive enzyme functions, representing a significant advancement in formulating diets crucial for successful octopus farming that may help fully understand its physiology.

## INTRODUCTION

*Octopus maya* (*O. maya*) is an endemic species of the Yucatan continental shelf, supporting one of the most important octopus fisheries worldwide with an annual production ranging from 8 000 to 20,000 tons (t) (Markaida et al., 2017). Due to its commercial value, it is one of the top five national fisheries in Mexico (Arreguín-Sánchez et al., 2000). The controlled cultivation of aquatic organisms (aquaculture) presents a viable solution to supplement fisheries and ensure a steady supply of octopus while mitigating the impact on natural populations. However, the successful transition from traditional fisheries to aquaculture requires careful consideration of various factors, with nutrition being a critical aspect. Thus, feeding is a key factor in aquaculture, directly affecting growth, survival, maturation, reproduction, health (Abdollahpour et al., 2022; Vijayaram et al., 2023), and consequently, performance. In the case of cephalopods, our knowledge is still in the early stages of finding feeds with appropriate nutritional composition (Domingues et al., 2007; Rosas et al., 2011), posing a challenge for large-scale commercial production.

Research on artificial diet development for cephalopods has focused on understanding nutritional needs regarding total and class lipids, protein content, minerals, and flours. Various raw materials (other cephalopods, fish, and crustacean species); binders (alginate, gelatin); and raw material preparation (fresh, dried or lyophilized) have recently been evaluated for preparing artificial diets (Cerezo-Valverde et al., 2008; Estefanell et al., 2013; Garcia et al., 2011; García-Garrido et al., 2010, 2013; Hamdan et al., 2014; Morillo-Velarde et al., 2011, 2012, 2015; Querol et al., 2015a,b; Cerezo-Valverde et al., 2012, 2013).

Feeds based on natural diets (e.g. crustaceans, fish, and mollusks), wet diets (paste mixtures of natural prey or dried), and formulated diets (agglomerated mixtures of wet and/or dry ingredients with different additives) for growing octopuses have been documented for the common octopus *Octopus vulgaris* (*O. vulgaris*), octopus *O. maya*, monkey-faced octopus *Octopus mimus* (*O. mimus*), and *Enteroctopus megalocyathus* (*E. megalocyathus*) (Gutiérrez et al., 2015; Martínez et al., 2014; Rodríguez-González et al., 2015; Zúñiga et al., 2011).

For octopuses, proteins are the main metabolic substrate characterized by a natural diet primarily based on crustaceans, mollusks, and fish (Alejo-Plata et al., 2018; Estefanell et al., 2013). Previous studies have demonstrated the importance of crustaceans in octopus diets, showing that up to 19 crustaceans species can be found in the diet of *O. vulgaris* wild paralarvae (Roura et al., 2012) and 22 in the diet of *O. maya* juveniles (Markaida, 2023). Thanks to research on physiological digestion, a semi-wet paste based on squid and crab meat was recently developed as a successful diet for *O. maya* juveniles and adults (Gallardo et al., 2020; Martínez et al., 2014; Tercero et al., 2015). By comprehending how efficiently juvenile octopuses digest certain food components, such as proteins from squid and crab meat, researchers could design a diet that maximized nutrient absorption and supported optimal growth. This approach ensured that the diet was not only nutritionally balanced but also aligned with the digestive capabilities of the octopuses, thereby enhancing its effectiveness in promoting growth and development. With this diet, wild females were successfully acclimated (Caamal-Monsreal et al., 2015; Tercero et al., 2015), and juveniles, and pre-adults grew to spawning, with reported growth rates of 3.04% per day in juvenile *O. maya* (Martínez et al., 2011, 2014). The diet was based on the digestive capacity of juvenile octopuses and successfully used to cultivate offspring until they reached 250 g of body weight, as demanded by gourmet market (Rosas et al., 2014). Regarding digestive capacity, it denotes the digestive system's efficiency in breaking down food into absorbable nutrients. It varies among individuals and is influenced by factors like diet, health, and digestive organ function (Ayala-Berdon and Schondube, 2011; Saldaña-Vázquez et al., 2015). Although this diet has been well accepted by juveniles the remaining challenge is to develop animal feed with cheaper ingredients, such as fish scraps from fisheries activities. Historically, diets formulated with fish scraps have been observed to yield lower growth rates than those made with squid-crab paste (Pérez et al., 2006). This difference has been attributed to factors such as lower acceptability (Pérez et al., 2006), conversion index (Domingues et al., 2010), and probably digestibility (Martínez et al., 2014).

To design and develop formulated feed for cephalopods, understanding their digestive physiology is essential. Given the carnivorous feeding habits of octopuses, proteolytic enzymes play a key role in their digestive process. Among these enzymes are trypsin and chymotrypsin, which originate from both the posterior salivary gland (Grisley et al., 1996; Morishita et al., 1974; Omedes et al., 2022; Suzumura et al., 2023) and the digestive gland. These enzymes are among the most widely studied in cephalopods (Mancuso et al., 2014; Martínez et al., 2011, 2012; Pereda et al., 2009; Rosas et al., 2011). In comparison to proteases, studies on carbohydrases and lipases in cephalopods are limited to *Octopus cyanea* (Boucher-Rodoni, 1973; Omedes et al., 2022), *Eledone cirrosa* (Boucher, 1975), *O. vulgaris* (Boucher-Rodoni and Boucaud-Camou, 1987; O'dor et al., 1984), *O. maya* (Aguila et al., 2007; Gallardo et al., 2017; Moguel et al., 2010), *O. bimaculoides* (Ibarra-García et al., 2018; Solorzano et al., 2009) and *O. mimus* (Linares et al., 2015). Analysis of digestive enzymatic activity in octopuses has been considered an indicator of their ability to digest different types of food at various life stages, breeding conditions (Farías et al., 2016), and in response to other environmental factors (Uriarte et al., 2016).

Distinct from other digestive enzymes previously mentioned (proteases, carbohydrases, and lipases), acidic enzymes (such as acid phosphatases, cathepsins B, D, H, and L) were first observed in the crop, stomach, and digestive gland (DG) of *O. vulgaris* (Morishita et al., 1974). Subsequent studies by Martínez et al. (2011) and Linares et al. (2015) indicate the presence of acidic enzymes not only in *O. vulgaris* but also in *O. maya* and *O. mimus*. Additionally, these enzymes have been observed in other cephalopod species, such as squid and cuttlefish (Cardenas-Lopez and Haard, 2005, 2009; Perrin et al., 2004), suggesting their significant role in cephalopod digestion. In a partial characterization of *O. maya* digestive enzymes in gastric juice (GJ) (i.e. the fluid found in the stomach but not secreted by the stomach) and DG (Martínez et al., 2011), cathepsin D was found to be inhibited by 18 and 72% in GJ and DG, respectively, and requires an acidic environment to develop its maximum activity. Recently, Fernández-Gago et al., (2018) demonstrated that digestive enzymes are synthesized in digestive gland, where are send, as gastric juice, to digestive system.

This observation underscores the importance of acidic enzymes in the digestive process of this octopus species, as previously demonstrated by Morishita et al. (1974). However, this family of enzymes (cathepsin) has shown sensitivity to the biochemical structure of ingested proteins (Santé-Lhoutellier et al., 2008).

Given the protein-rich diet of octopuses (Cerezo-Valverde et al., 2012; Rosas et al., 2013), it is imperative that the enzymes involved in protein digestion exhibit high efficiency and rapid responsiveness to food arrival in the digestive tract. Consequently, the most extensively researched enzymes in cephalopods to date are those of a proteolytic nature, including trypsin, chymotrypsin (Boucaud-Camou and Boucher-Rodoni, 1983; Martínez et al., 2011), and cathepsins (Barrett and Kirschke, 1981; Gallardo et al., 2017; Ibarra-García et al., 2018).

Therefore, the hypothesis is that acidic enzymes, such as cathepsins, could initiate and catalyse reactions more efficiently and rapidly than alkaline enzymes as chymotrypsin. This phenomenon suggests an evolutionary adaptation by *O. maya*, where acidic enzymes could possess biochemical characteristics that allow them to have lower activation energies, providing a more agile response to the arrival of protein-rich foods in the digestive tract. This adaptation would align with the requirements of a predatory diet with a high demand for proteins.

The results obtained so far indicate a synchronization between the pulses of DG enzymes and GJ enzymatic activity. In *O. maya*, two pulses were observed (20-80 and 80-180 min), while only one (80-180 min) was observed in *O. mimus* enzymatic activity, suggesting strong differences in digestive dynamics between species (Linares et al., 2015). These differences could be due to the different environmental temperatures in the habitat of each species, with more frequent enzyme release in tropical (e.g. *O. maya*) than in subtropical or temperate species (*O. mimus*). Therefore, temperature could be regulating the entire digestive activity, including ingestion rate, chyme formation, intracellular digestion, and enzyme production.

Given all of the above, exhaustive studies should be performed to fully understand *O. maya* digestive physiology. The relationship between enzymatic activity and the octopus nutritional status, as well as its impact on different life stages and breeding conditions underscores the importance of enzymatic characterization to assess and improve diets. This profound knowledge of digestive physiology will not only contribute to overcoming current obstacles in commercial production but also provide a solid foundation for the sustainable development of the octopus fishery, ensuring the conservation of this species.

## RESULTS

### Determination of the optimal temperature for the activity of acid and alkaline proteases

**Fig. 1.**
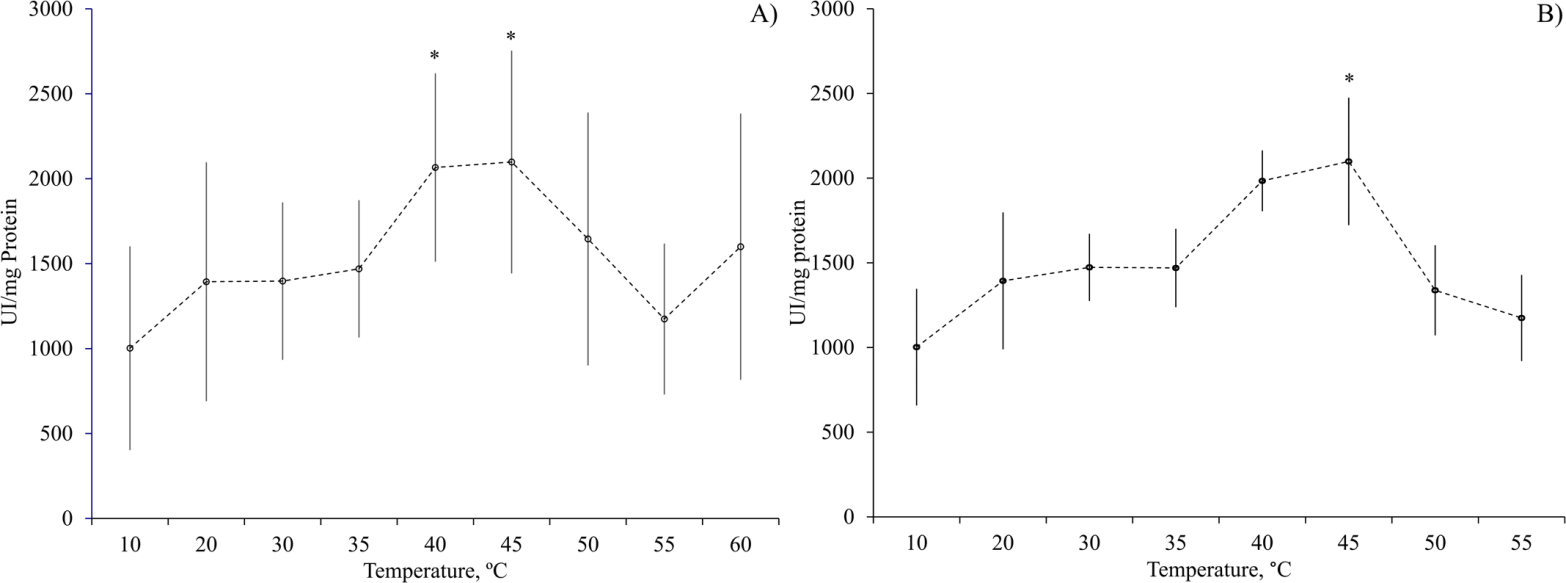
**Effect of temperature (°C) on acid proteases activities of (A) gastric juice (GJ) (at pH 6.0) and (B) digestive gland (DG) (at pH 3.0) of *O. maya*.** Asterix indicates statistical differences at *P*<0.05 level. Data show mean±s.e. *N*=6.

In the experiments aimed at identifying the optimal temperature for the activity of GJ acid proteases, low activities were noted at temperatures ranging from 10 to 35°C, with a noticeable surge in the activity observed at 40 and 45°C (*P*>0.05; Fig. 1A). In regard to the acid digestive enzymes in the DG, their peak activity showed at 45°C (*P*<0.05; Fig. 1B). Notably, a rise in activity was also observed from 20 to 30°C, suggesting the potential presence of enzymes with optimum activity within that temperature range (Fig. 1B).

**Fig. 2.**
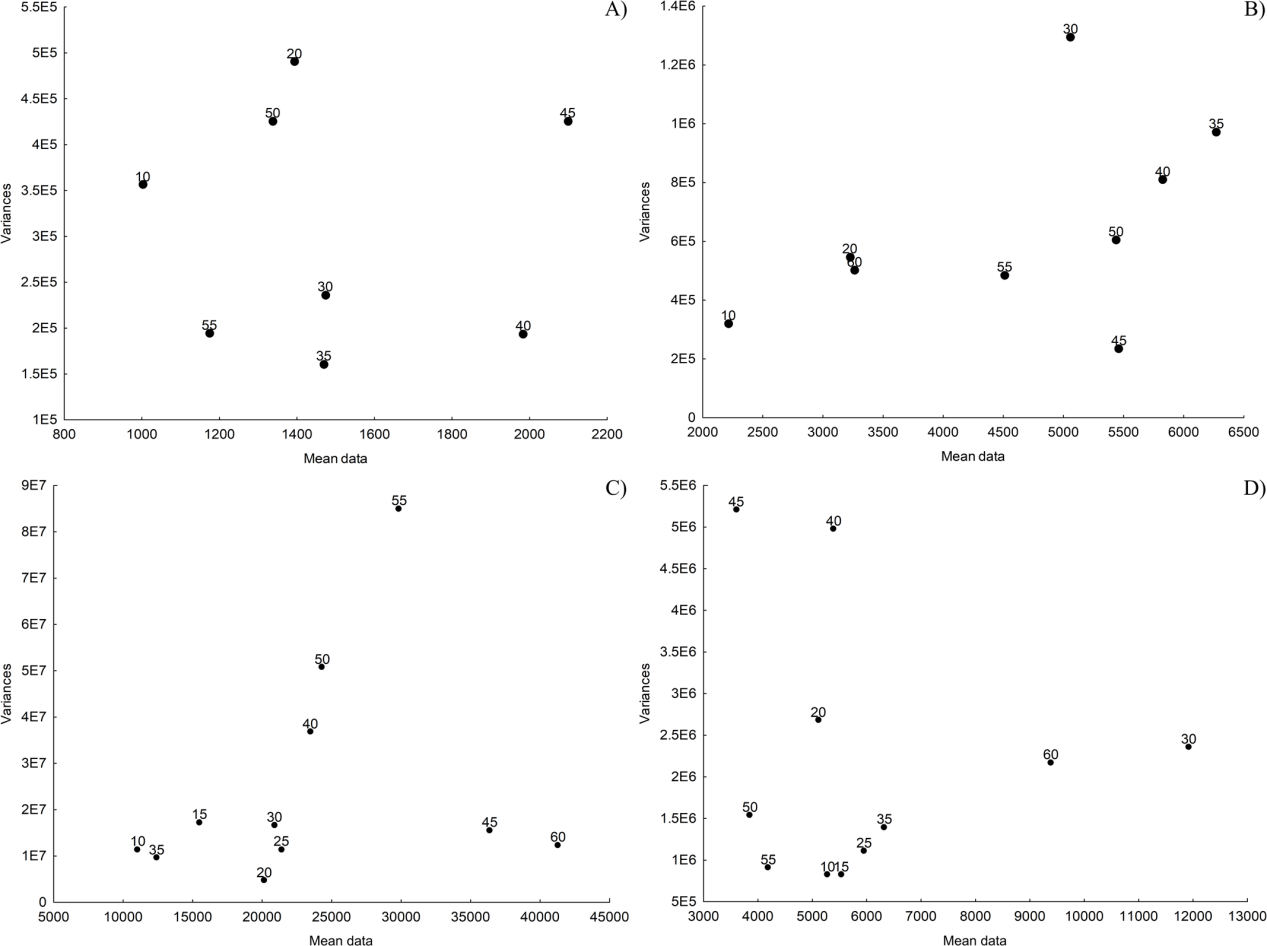
**Variance-mean data relationship.** Dispersion of variances plotted against the mean data values of data to enzymes activities corresponding to each temperature in the assessment of *Octopus maya* gastric juice (GJ) [(A) acid proteases and (B) alkaline proteases] and digestive gland (DG) [(C) acid proteases and (D) alkaline proteases]. The x-axis displays the mean values of data in U mg protein^−1^, while the y-axis represents the corresponding variances for each average. Each point on the graph represents the mean value at each analyzed temperature.

The variance versus mean data graphs were used to establish which of the temperatures with maximum activity would be chosen as the optimal one for evaluation of acid and alkaline protease activity of *O. maya* gastric juice and digestive gland. The temperature of 45°C turned out to promote the greatest activity. However, at this temperature GJ enzyme activity was more dispersed than that observed at 40°C (Fig. 2A). Taking this into account, the temperature of 40°C was chosen as the optimal one for the evaluation of *O. maya* GJ acidic enzymes.

The GJ alkaline protease enzymatic activity was higher at 35°C (*P*<0.05; Fig. 2B). The DG acid proteases shown a maximum activity at 45°C (*P*<0.05; Fig. 2C). The maximum activity of DG alkaline proteases was obtained at 30°C (Fig. 2D). This activity was shown as a single, well-defined and highly significant peak (*P*<0.05).

**Fig. 3.**
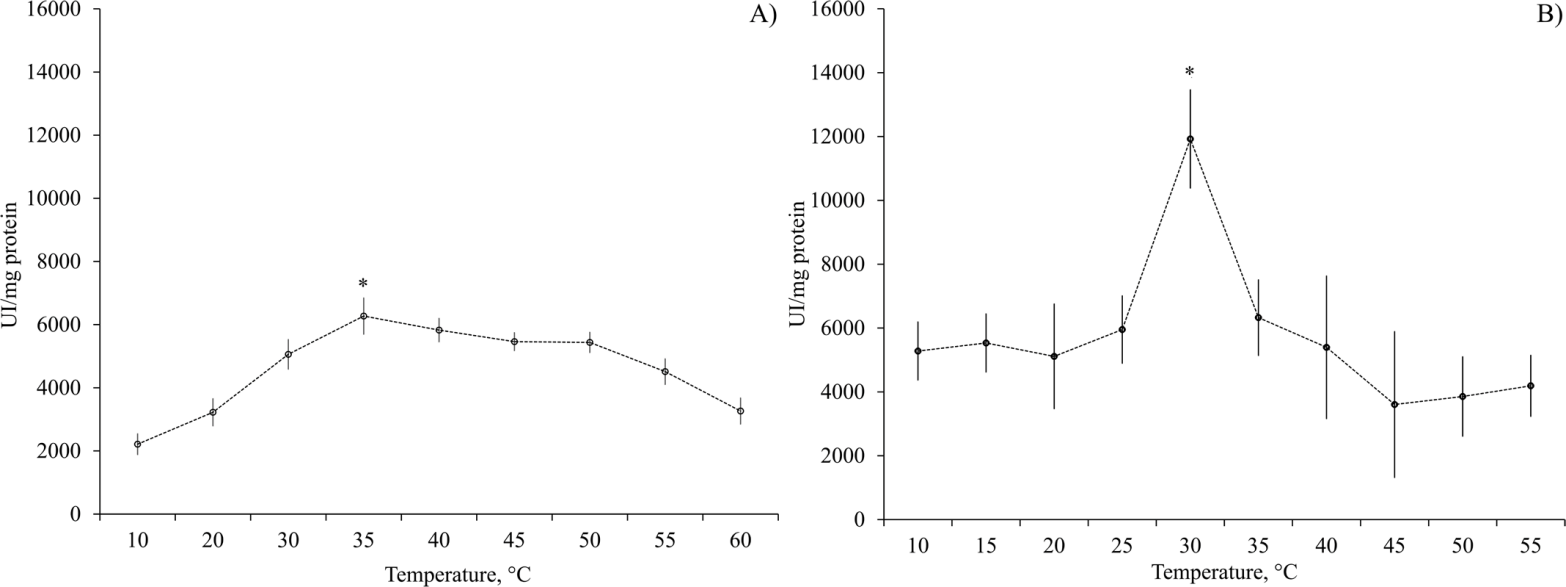
**Effect of temperature (°C) on alkaline proteases activity (at pH 8) of (A) gastric juice (GJ) and (B) digestive gland (DG) of *O. maya*.** Asterix indicates statistical differences at *P*<0.05 level. Data show mean±s.e. *N*=6.

When the enzymatic activity results of alkaline proteases (ranging from 2213 to 11923U mg protein^−1^) (Fig. 3) are compared with those of acidic proteases (ranging from 1002 to 2098U mg protein^−1^) (Fig. 1), alkaline proteases evidently show notably higher activity levels. In particular, the DG alkaline proteases displayed the highest activity value recorded, reaching 11293U mg protein^−1^.

### Determination of the optimal mix of enzymes of GJ and DG concentration

**Fig. 4.**
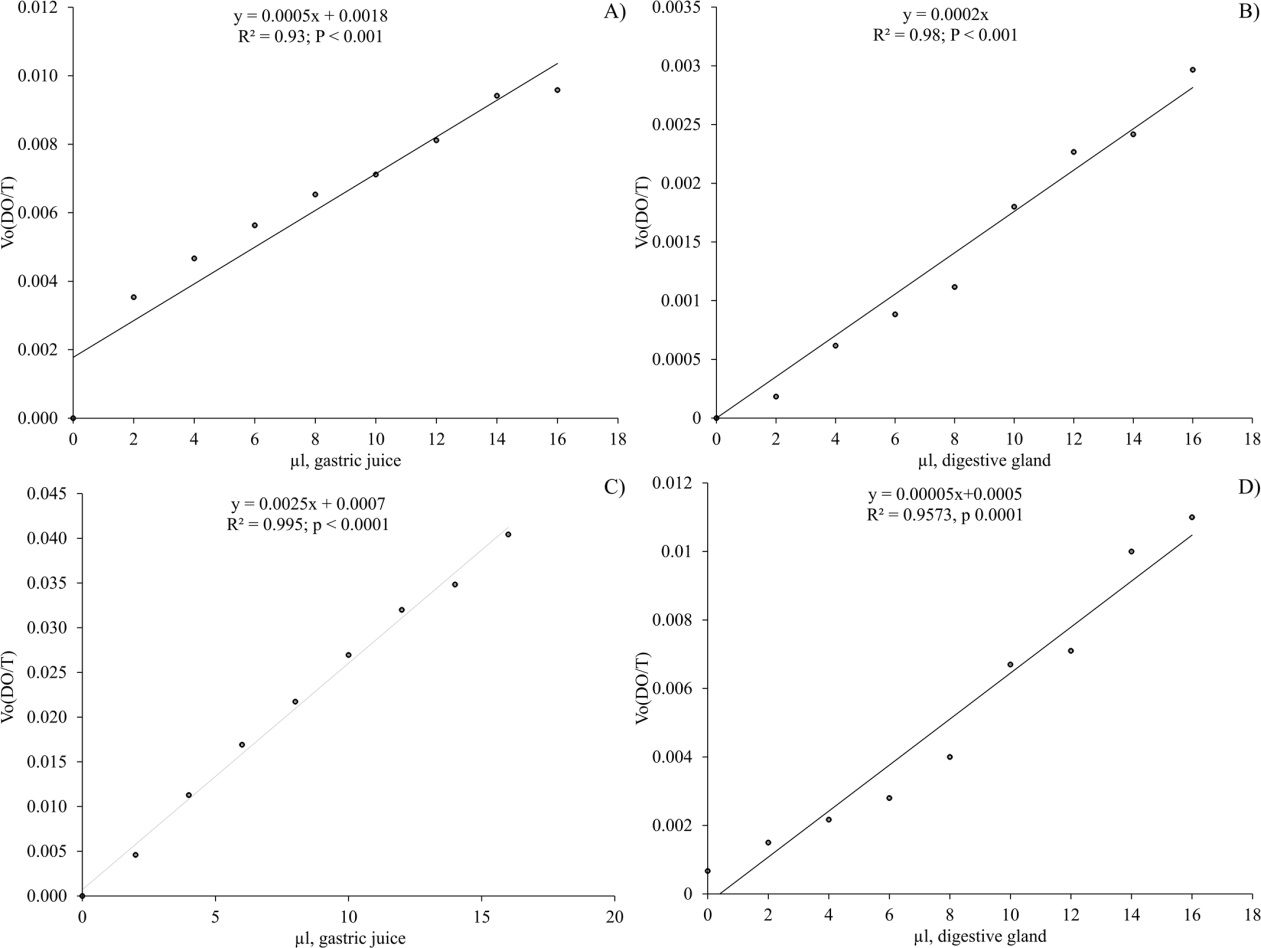
**Evaluation of enzymatic variation activity in response to different enzyme concentrations at the identified optimal temperatures.** The effect of enzyme concentration from gastric juice (GJ) and digestive gland (DG) on *O. maya*'s acid and alkaline protease activity, tested at optimal temperatures determined for enzymes from GJ and DG is shown as (A) acidic proteases from GJ at 40°C; (B) acidic proteases from DG at 45°C; (C) alkaline proteases from GJ at 35°C; (D) alkaline proteases from DG at 30°C. Each graph of linear regression displays R^2^ and *P*-values.

Once the optimal temperature was obtained, the tests were carried out to determine the enzyme concentration that maintains a constant activity, observing that from 2 to 16 µl of GJ or DG extract the enzyme activity increased proportionally with the amount of enzyme tested (Fig. 4).

The slope values of the relationship between activity and enzyme concentration were 0.0005 (µL GJ) (Fig. 4A) and 0.0002 (µL of DG homogenate) (Fig. 4B) for acidic proteases; 0.0025 (µL GJ) (Fig. 4C) and 0.00005 (µL of DG suspension) for alkaline proteases (Fig. 4D). Taking the above into account, a minimum concentration suitable for *O. maya* digestive enzyme evaluation was considered to be 2 µl for both the GJ analysis and the DG homogenate.

### Activation energy

**Fig. 5.**
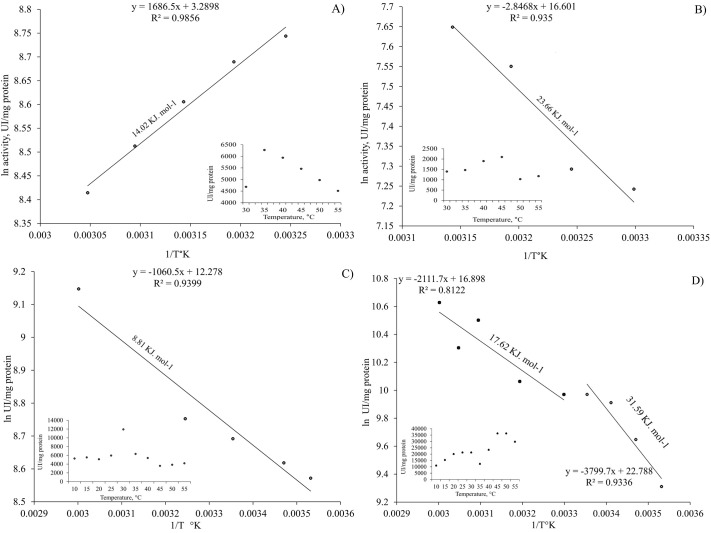
**Arrhenius plot analysis for determining activation energy.** This figure presents Arrhenius plots used to calculate the activation energy of both acidic and alkaline enzymes found in *Octopus maya* gastric juice (GJ) and digestive gland (DG). The distinct panels (A, B, C, and D) delineate the analysis for each enzyme type and tissue location, (A) alkaline enzyme of GJ; (B) acidic enzyme of GJ; (C) alkaline enzyme of DG; (D) acidic enzyme of DG.

The activation energy (Ea) of all the tests was calculated from the results obtained (Fig. 5), of which the highest Ea value was obtained for the peak of maximum activity recorded at 45°C in the acidic proteases from the DG (Fig. 5D, 31.59 KJ mol^−1^), while the lowest one was at 30°C for the alkaline DG proteases (Fig. 5C, 8.81 KJ mol^−1^). Intermediate Ea values were obtained for alkaline and acidic proteases of the gastric juice (Fig. 5A, 14.02 KJ mol^−1^ and B, 23.66 KJ mol^−1^). Similarly, an Ea intermediate value was obtained for the maximum activity of the range from 20 to 30°C of the acidic enzymes in DG for which two values of Ea were calculated (Fig. 5D, 17.62 KJ mol^−1^).

### Effect of inhibitors on cathepsins activity

**Fig. 6.**
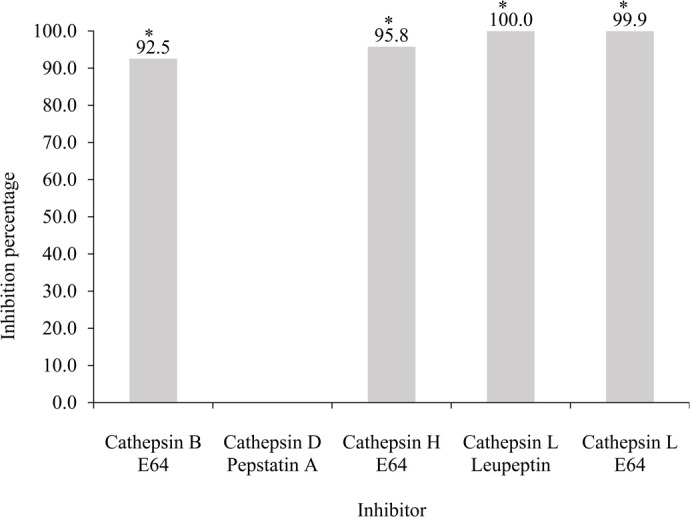
**Inhibition percentage by cathepsin enzyme inhibitors present in *Octopus maya* gastric juice (GJ): cathepsins B by E64, D by pepstatin A, H by E64 and L by E64 and leupeptin inhibitions.** Asterisks indicate significant difference (*P<*0.05). Inhibition percentage=[(D1 – D2)/D1×100] where D1=negative control, D2=treatment (Pineda-Suazo et al., 2021; Worasatit et al., 1994). The summary of the values used for the inhibition percentage and statistical analysis is given in Table S1.

The inhibition of B, H, L, and D cathepsins was verified by measuring their activities in the presence of E64, leupeptin and pepstatin A inhibitors. Cathepsin H and L activities were significantly reduced by 95.8 and 99.9%, respectively following treatment with E64 inhibitor. Cathepsin L showed a complete enzymatic activity reduction by leupeptin. Cathepsin D did not show enzymatic activity reduction by pepstatin A inhibtor. Cathepsin B showed a 92.5% activity reduction by E64 (Fig. 6).

## DISCUSSION

The digestive anatomy of cephalopods, like other mollusc species, has been extensively characterized (Andrews et al., 2022; Omedes et al., 2022; Sykes et al., 2017). It comprises muscle tissue, anterior and posterior salivary glands, digestive gland, and appendices (Andrews et al., 2022; Omedes et al., 2022). When food is ingested, it mixes with saltwater and secretions from the salivary glands, which include serine proteases, proteins, and amylase (Fingerhut et al., 2018; Pech-Puch et al., 2016). This mixture is then transported to the anterior stomach for the initial stages of digestion (for details see Andrews et al., 2022).

To better understand the enzyme activity in *O. maya*, we conducted tests to determine the enzyme concentration that maintains constant activity. Our observations revealed that the enzyme activity increased proportionally with the amount of enzyme tested, ranging from 2 to 16 µL of GJ or DG extract (Fig. 4). This indicates a direct correlation between enzyme concentration and activity, providing insights into the optimal conditions for enzyme function in the digestive processes of *O. maya*. Our findings align with the observations of Andrews et al. (2022), who noted that digestive juice enzyme activity can be highly variable, especially with a mixed diet. This variability underscores the importance of understanding enzyme concentration and activity to optimize the digestive efficiency in cultured species like *O. maya*. Additionally, a recent histochemical study of the digestive tract of *O. vulgaris* by Fernández-Gago et al. (2018) detected no digestive enzyme presence in the digestive tract epithelium. This finding supports the view that chyme formation during digestion is predominantly driven by digestive enzymes secreted from the digestive gland (DG), with a minor contribution from salivary enzymes. Consequently, the changes occurring as food passes along the digestive tract are mainly due to the pulses of enzyme production in the DG during the digestion phases, as described in previous cephalopod studies (Linares et al., 2015; Martínez et al., 2011). The time required to digest a meal varies depending on the species and ambient temperature. Our study did not focus on correlating enzyme activity with the habitat temperatures of *O. maya*; however, understanding the enzyme kinetics and optimal conditions for enzyme function remains crucial. These insights can significantly contribute to the formulation of effective diets and the successful cultivation of *O. maya* by ensuring that the enzymes involved in digestion are operating at their most efficient levels.

Cardenas-Lopez and Haard, 2005 documented the existence of proteinases extracted from the jumbo squid hepatopancreas (*Dosidicus gigas*), primarily of the cysteine type. These proteinases exhibited notable activity towards a substrate specific to cathepsin L. These observations were confirmed in the present study by using enzyme inhibitor leupeptin, E64, and pepstatin, thereby providing conclusive evidence for the presence of cathepsins in *O. maya* digestive juice.

Leupeptin is a broad-spectrum inhibitor of serine and cysteine proteases widely used in global protein purification workflows, showing inhibitory activity against A, B, and D cathepsins (Aoyagi et al., 1969). On the other hand, E64 is a potent irreversible inhibitor for various cysteine proteases, such as papain, B, H and L cathepsins (Katunuma and Kominami, 1995; Vidal-Albalat and González, 2016). While pepstatin A, is a specific inhibitor of aspartyl proteases such as cathepsin D, cathepsin E, pepsin, and renin (Zhang et al., 2000). Accordingly, these inhibitors have been widely used to identify and characterize proteases and assess their roles in biological processes (Barrett and Kirschke, 1981; Monteriez et al., 1994). To determine the potential involvement of cysteine-proteinases in *O. maya* digestion, the effects of the proteinase inhibitors leupeptin, pepstatin A and E64 were investigated. Our findings revealed that E64 inhibitor reduces enzyme activity up to 99.94%, while leupeptin achieves a reduction of 99.96%. These strong data suggest that cathepsins B, H, and L play an essential role in *O. maya* digestive processes. However, assays using the inhibitor pepstatin A did not inhibit enzymatic activity, this differs from what was reported by Martínez et al., in 2011. They observed a reduction in enzyme activity of 70 and 20% in hepatopancreas and GJ of *O. maya*, respectively when protease inhibitor Pepstatin A was used. Given the discrepancy between our findings and those of Martínez et al., (2011), we decided to perform a bioinformatic analysis in search of cathepsins, pepsin, and other digestive enzymes in the genome and transcriptome of *O. maya* (the results of this study will be published soon). During this process, we found no evidence supporting the presence of cathepsins D, E, or pepsin in *O. maya*. These bioinformatics results reinforce our conclusion about the absence of these enzymes in the species.

To conduct a more comprehensive analysis of the enzymatic activities of both acidic and alkaline proteases in *O. maya*, we analyze their activation energy. Our primary objective was to characterize these enzymes and shed light on their overall biochemical properties and distinctions for the first time in octopus species. Therefore, in our experiments, we analyzed the proteases of the digestive gland and gastric juice across a pH range of 3, 6, and 8, and temperatures spanning from 10 to 60°C.

Understanding the enzymatic activity of *O. maya* at various temperatures, including those beyond its normal physiological range, is crucial for assessing the species’ response to environmental stress. This research illuminates the metabolic adaptability of *O. maya* by showing how its enzymes perform under different thermal conditions. It has been demonstrated that higher temperatures within a species’ natural range increase metabolic demand (Seibel and Deutsch, 2020). By studying these activities, we can predict the octopus's response to unusual thermal stress or environmental changes, which is vital for both natural resilience and aquaculture practices. This insight is essential for overcoming current challenges in commercial production and fostering sustainable development in aquaculture.

Turk et al. (1993) evaluated the effect of temperature (5-37° C) at pH 7.4 in L cathepsin showing an activation energy of 174.7 kJ mol-1 (41.8 kcal mol-1). In 1994, Turk et al. (1994) evaluated cathepsin B in the temperature range of 5-30°C at pH=8.0, using 50 mM Hepes buffer, containing 100 mM NaCl and 1 mM EDTA. Under these conditions an activation energy of 183.5 kJ mol-1 was calculated from the Arrhenius plot slope. Both results (L and B cathepsins) are comparable between them. Additionally, both values are also very close to that found for the staphylococcal nuclease alkaline-pH-induced unfolding (195.0 KJ mol^−1^) (Chen et al., 1991). In our experiments, the proteases of the digestive gland were analyzed at pH 6 and 8, covering a range of temperatures from 20 to 45°C. The highest *Ea* value was observed at 45°C (31.59 KJ mol^−1^), intermediate values (17.62 KJ mol^−1^) at 20-30°C and lowest value (8.81 KJ mol^−1^) at 35°C. The variability in *Ea* values among various conditions and enzymes could reflect the complexity of *O. maya* digestive processes. Since these results contrast with those previously obtained in studies under similar conditions for cathepsins L and B (Chen et al., 1991; Turk et al., 1993, 1994), they suggest the possible presence of digestive enzymes other than these in the digestion of *O. maya*.

An interesting emerging observation was that alkaline enzymes had a lower activation energy compared to acidic enzymes. This finding led us to question our previous assumptions about the predominant enzymes in *O. maya* digestion, as we had initially assumed that acidic enzymes, such as cathepsins, would have a lower activation energy due to the high-protein carnivorous diet characteristic of this species. However, it was not the case (Fig. 4), prompting us to reconsider our hypotheses. The proposal is that in addition to cathepsins, alkaline enzymes, such as alkaline phosphatase could play a role in the absorption of amino acids and, in turn, could be involved in cathepsin activation. Possibly, octopuses absorb the enzymes present in their prey at the beginning of external digestion (Boucaud-Camou and Roper, 1995). If these preys (mostly crustaceans) contain alkaline enzymes, such as trypsin (Córdova-Murueta et al., 2003; Souza Buarque et al., 2009), upon entering the digestive tract they could activate the zymogens stored in the anterior stomach, preparing them for the chyme arrival (Boucaud-Camou and Roper, 1995; Linares et al., 2015). This activation would be crucial since zymogens remain inactive until food arrives.

Furthermore, alkaline proteases may have evolved to function efficiently in both acidic and alkaline environments, which could be due to pH variability in the gastrointestinal tract that would require the enzymes to be active under different conditions. Therefore, alkaline proteases could have evolved a structure and catalytic mechanism that allows them to have a lower activation energy in an acidic environment as the stomach. This situation could be supported by the results found by Martínez et al. (2011), where activity was observed at pH 7 and 8 in GJ, suggesting the presence of alkaline enzymes. When studying the activity of digestive enzymes was studied throughout the digestive tract and over digestion time in *O. maya* and *O. mimus*, Linares et al. (2015), observed alkaline enzyme activity peaks, which were interpreted as changes in pH as digestion progressed. Initially, the pH was acidic, and as acidic enzymes degraded proteins, OH- ions were released, facilitating the activation of alkaline enzymes present in the digestive tract. Then, a shift was observed and the activity of the alkaline enzymes decreased, followed by an increase in the acidic enzyme activity, which was interpreted as the pulse result of enzymes coming from the digestive gland that “boost” digestion by sending “fresh” acidic enzymes to complete it.

Talking about the role of phosphatases as a cofactor to activate proteases, such as trypsin and chymotrypsin, it might be indicative that specific interactions between alkaline proteases and other components present in the stomach could decrease their activation energy. For example, certain cofactors or ions present in the acidic environment of the stomach could stabilize the structure of alkaline proteases and facilitate their catalytic activity, thus reducing their activation energy.

Finally, the activity of proteases, both acidic and alkaline, should be subjected to regulation by other factors, such as post-translational modifications or interactions with other proteins present in the stomach environment. These regulations could lead to a reduction in the alkaline protease activation energy compared to acidic proteases in certain specific contexts. Post-translational modifications have the potential to affect enzymatic activity by modifying the three-dimensional protein structure, which in turn can influence its ability to interact with an affinity for its substrate. These changes could lead to a decrease in the activation energy required to initiate the enzymatic reaction, suggesting that alkaline proteases could be more efficient and active in an acidic environment as the stomach. Therefore, more exhaustive research should help to better understand the set of enzymes involved in *O. maya* digestion.

### Conclusion

Mollusk digestive anatomy – particularly cephalopods like *O. maya* – has been extensively studied, revealing a complex system involving muscle tissue, salivary and digestive glands, and appendices (for more details see Andrews et al., 2022; Linares et al., 2015; Lobo-da-Cunha, 2019). Our investigation confirmed the presence of various enzymes, including cathepsins B, H, and L, in *O. maya* digestive system. Through the use of enzyme inhibitors like leupeptin, E64, and pepstatin A, the significant role of cysteine proteases in *O. maya* digestion was demonstrated. Notably, we observed that alkaline enzymes were observed to be a lower activation energy compared to acidic enzymes, challenging previous assumptions and suggesting a potential role for alkaline phosphatase in amino acid absorption and cathepsin activation. This finding implies a sophisticated evolutionary adaptation to variable pH conditions in the gastrointestinal tract, supported by enzyme activity observations at different pH levels. Moreover, the presence of specific interactions and regulatory mechanisms involving phosphatases and other proteins in the stomach environment may contribute to the observed differences in activation energy between acidic and alkaline proteases. Overall, further comprehensive research should warrant to fully elucidate the diverse enzyme repertoire involved in *O. maya* digestion and its implications for evolutionary adaptation and ecological dynamics.

## MATERIALS AND METHODS

### Sample collection

A group of *O. maya*, six wild adults (500±250 g live weight) obtained from the coast of Sisal, and San Felipe, Yucatán, Mexico were kept in acclimation for 10 days and fed *ad libitum* with fresh crab (*Callinectes* spp), maintained at 24°C and 34 UPS in a recirculatory and aerated seawater system, coupled to 20 and 5 µm cartridge filters. After the acclimation period, gastric juice was obtained from animals fasted for 12 h, stimulating gastric secretion by placing bags with dead crabs.

These bags allowed the octopuses to be in contact with the prey without having the opportunity to ingest them for a period of 20 min. Subsequently, the animals were euthanized to extract the GJ produced and accumulated in the crop. Therefore, animals were placed in cold sea water maintained at 15°C for 5 min and were after wards euthanized by cutting between the eyes into the brain. Subsequently, the mantle was cut longitudinally to locate the stomach, which was clamped at both terminal ends in order to extract the juice accumulated between the distal portion of the cecum and the crop. Digestive glands sections were dissected and frozen in liquid nitrogen and then stored at −80°C until analysis. At the same time the volume of the chyme accumulated was measured (±1 ml) and immediately frozen in liquid nitrogen and stored at −80°C until analysis.

Experimental procedures were approved by the Ethics and Scientific Responsibility Commission of Faculty of Sciences at National Autonomous University of Mexico (CEARC/Bioética/25102021). This commission followed the European directrices used in European Union related with the use of cephalopods as experimental animals. Taking into consideration that until now there is no directrices to Page 12/25 the cephalopods embryos, this study was designed to generate a base line to generate knowledge useful to propose directrices to use embryos as experimental animals.

### Evaluation of the physicochemical and biochemical conditions of GJ and DG enzymes

#### Protein quantification and enzyme activity assays

Total soluble protein concentration was determined (Bradford, 1976) using serum bovine albumin as standard. Acid and alkaline proteases were characterized in 96-well plate for reader using chemicals by Anson methods (Anson, 1938), and adjusted to microplate reader. Acid proteases activity was assayed at pH 6.0 (GJ) and pH 3 (DG) using hemoglobin (Sigma, H2625) (1%) as substrate; alkaline protease activity was assayed at pH 8 and using casein (1%) as substrate. Previous studies showed that the alkaline proteases of *O. maya* has its higher activities at pH 8, while acidic proteases have its maxim activities at pH 3 in digestive gland and in pH 6 in gastric juice (Martínez et al., 2011). Briefly, 20 µl of the enzyme extract (dilution 1:10) was mixed with 500 µl of stauffer buffer; 500 µl of freshly prepared substrate in stauffer buffer at the corresponding pH and incubated at 37°C for 10 min. The reaction was stopped by adding 500 µl of 20% (w/v) trichloroacetic acid (TCA Sigma, T6399) and cooling for 15 min at 4°C. The precipitated undigested substrate was separated by centrifugation at 13,370 ***g*** for 15 min. The supernatant absorbance was measured spectrophotometrically at 280 nm against the substrate without enzyme extract (blank) (see details in Martínez et al., 2011). All determinations were done in triplicates and included blanks, which consisted of stauffer buffer, substrate and TCA without enzyme extracts (Table 1). Blanks were incubated as mentioned earlier and read at each pH. To establish the possible acid enzyme (cathepsin D; Cardenas-Lopez and Haard, 2009) presence, the enzymatic extracts were incubated with pepstatin A (Sigma, P5318), 1 mM dissolved in dimethylsulfoxide (DMSO Sigma, D5879) as aspartic proteinase inhibitor at pH 3 (DG) and pH 6 (GJ). This enzyme activity assay was used for all the following experiments.

**Table 1.**
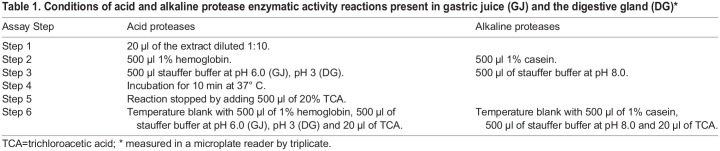
Conditions of acid and alkaline protease enzymatic activity reactions present in gastric juice (GJ) and the digestive gland (DG)*

#### Determination of the optimal temperature for the acid and alkaline protease activities

The experimental temperatures in this phase of the present study were set from 10 to 60°C with intervals of 10°C between them. To fine-tune maximum activities, 5°C intervals were also included when necessary. To do that, gastric juice, and digestive gland samples (with their buffer and specific substrate) were incubated for 10 min at each experimental temperature, placing the samples in a thermoregulated bath. After, the reaction was stopped using TCA (20%) centrifuged (13,370 ***g***) and read in a microplate by triplicate, as was described above.

#### Determination of the activation energy

The activation energy was calculated using the Arrhenius equation, which relates the reaction rate of a chemical reaction to temperature. The Arrhenius equation is expressed as: 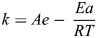 Where: *k* is the reaction rate constant. *A* is the intercept of the relationship between the logarithm of enzyme activity versus inverse of temperature expressed in Kelvin degrees, representing the frequency of effective collisions between molecules. *Ea* is the activation energy, representing the minimum energy required for the reaction to occur. *R* is the gas constant [8.314 J/(mol*K)]. *T* is the absolute temperature in kelvin. By plotting ln(*k*) versus 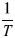, where *k* is the reaction rate and *T* is the temperature in kelvin, the slope of the resulting line on the graph is equal to 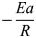, allowing the activation energy *Ea* to be calculated from the slope of the line. These plots elucidate the temperature dependence of enzyme activity and offer insights into the thermodynamic characteristics of enzymatic reactions in *O. maya*. Analysis of temperature dependency is depicted in each Arrhenius plot.

#### Determination of the optimal enzyme concentration

The experimental enzyme concentrations in the reaction mixture were established between the protein mg contained in a range of 2 to 16 µl of GJ or DG. To compare the results obtained, the experiments were carried out for both, the gastric juice and the digestive gland at 37°C and the previously determined optimal temperatures.

Evaluation of the activity and identification of cathepsins present in the gastric juice and digestive gland of *O. maya* using specific inhibitors.

#### Identification and evaluation of the activity of cathepsins in gastric juice

The standard method (Barrett and Kirschke, 1981) was followed to assess the activity of B, D, H, and L cathepsins in the GJ, which involved using cathepsin-specific substrates, as listed in Table 2. In this method we made use of a specific substrate MCA-Gly-Lys-Pro-Ile-Leu-Phe-Phe-Arg-Leu-Lys(Dnp)-D-Arg-NH_2_ [where MCA is (7-methoxycoumarin-4-yl)acetyl and Dnp is dinitrophenyl]. This substrate is digested by both, cathepsin E and cathepsin D and therefore can be used to detect cathepsin D (Yasuda et al., 1999). It was done considering that the digestive process starts in digestive tract, where cathepsins have the role to digest the food allowing the chyme, rich in nutrients, to reach the digestive gland where other digestive process occurs. For that reason, the evaluation of cathepsins were done only in gastric juice and not in digestive gland.

**Table 2.**
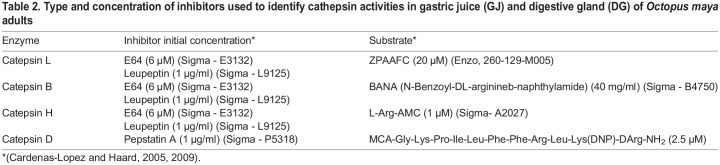
Type and concentration of inhibitors used to identify cathepsin activities in gastric juice (GJ) and digestive gland (DG) of *Octopus maya* adults

The reaction mixture was prepared with 50 µl of enzyme extract and 300 µl of buffer/specific substrate (pH 6.0) that was used (88 µM KH_2_PO_4_=11.96 gr./Lt, 12 mM Na_2_HPO_4=_1.704 gr./Lt, 1.33 mM EDTA=0.4950 gr./Lt, 2.7 µM cysteine=0.3267 gr./Lt). Subsequently, the mixture was pre-incubated at 40°C for 5 min. Then, 10 µl of the substrate in DMSO (see Table 2) was added and incubated at 40°C for 10 min. The reaction was stopped with 400 µl of the Fast Garnet GBC preparation in mersalic acid-Brij reagent, allowing it to rest to develop the color for 10 min. The reading was made in a spectrophotometer at 520 nm. The blank contained 50 µl of pyrogen-free water+300 µl of incubation buffer+10 µl of substrate+400 µl of the Fast Garnet GBC preparation in mersalic acid-Brij reagent (Cardenas-Lopez and Haard, 2005).

#### Effect of inhibitors on the activities of cathepsins

To identify the cathepsin percentage in the GJ, screenings with the cathepsin specific inhibitors E64 (Sigma - E3132), leupeptin and pepstatin A were carried out. The reaction mixture for all assays consisted of 50 µl of GJ and 50 µl of the corresponding inhibitor stock-solution (Table 2).

### Statistical analysis

Standard statistical techniques were used to analyze the data obtained in this research study. Significance was determined by Student's *t*-tests, independently by groups to evaluate the differences between the experimental conditions tested with both GJ and DG enzymes. One-way analysis of variance (ANOVA) to evaluate the differences of temperature dependency on *Octopus maya.* Additionally, a linear regression analysis was performed to examine the relationship between enzyme activity and substrates. All statistical analyses were performed using STATISTICA data analysis software (StatSoft, Inc. 2007, version 7. http://www.statsoft.com).

## Supplementary Material

10.1242/biolopen.060429_sup1 Supplementary information
