# Supplementary material for: Evaluation of Octopus maya enzyme activity of the digestive gland and gastric juice
Source: Biol Open. 2024 Sep 12;13(9):bio060429. doi: 10.1242/bio.060429 (PMC11413930; doi:10.1242/bio.060429)
Supplement: Supplementary information [file biolopen-13-060429-s1.pdf]

Table S1. Data for inhibition percentage calculation and t-tests of enzyme inhibitors of cathepsins B, D, H, and L.

A statistical significance level of $p < 0.07$ was established to evaluate the inhibition percentage by enzymatic inhibitors of cathepsins B, D, H and L, considering the variability of the enzymatic reactions and the relatively low sample size due to ethical restrictions. Inhibition was considered significant if *p* value was less than this threshold.

	Cathepsin B		Cathepsin D		Cathepsin H		Cathepsin L			
**Inhibitor**	**E64**	**Negative control**	**Pepstatin A**	**Negative control**	**E64**	**Negative control**	**Leupeptin**	**Negative control**	**E64**	**Negative control**
**S1**	212.62	2401.76	3.90	4.49	0.18	3.16	8.40734E-05	0.14	0.00012326	0.14
**S2**	281.95	3607.56	5.84	1.80	0.41	7.94	0.000129529	0.28	0.00015627	0.28
**S3**	150.09	2631.97	3.60	4.73	0.00	2.92	3.56706E-05	0.16	7.8348E-05	0.16
**S4**			5.02	5.72			0.000116201	0.26	0.00012848	0.26
**S5**			4.00				4.84518E-05	0.17	7.0059E-05	0.17
**Mean**	214.89	2880.43	4.45	4.19	0.20	4.67	8.27851E-05	0.20	0.00011128	0.20
**Std. Dev.**	65.96	640.15	1.22	1.67	0.20	2.83	0.00	0.06	0.00	0.06
**Inhibition percentage**	92.54		-6.31		95.76		99.96		99.94	
***p* value**	0.00		0.28		0.069		0.001		0.001	

Where **S** = sample reported in UI/mg protein

Inhibition percentage = [(**D1** – **D2**)/**D1** × 100] where **D1** = control, **D2** = treatment (Pineda-Suazo et al., 2021; Worasatit et al., 1994).
